# Supplementary material for: The Systems Biology Research Tool: evolvable open-source software
Source: BMC Syst Biol. 2008 Jun 29;2:55. doi: 10.1186/1752-0509-2-55 (PMC2446383; doi:10.1186/1752-0509-2-55)
Supplement: Additional file 1 — SBRT Archive. An archive of the current version of the Systems Biology Research Tool. [file 1752-0509-2-55-S1.zip › sbrt-1.4.0/doc/developers_guide/api/sbrt/ext/mma/algebra/MMaExprInterpretter.html]

MMaExprInterpretter


|  |  |  |
| --- | --- | --- |
| **Overview** \| **Package** \| **Class** \| **Use** \| **Tree** \| **Deprecated** \| **Index** \| **Help** | |  |
| **PREV CLASS**   NEXT CLASS | **FRAMES**    **NO FRAMES**     **All Classes** |  |
| SUMMARY: NESTED \| FIELD \| CONSTR \| METHOD | DETAIL: FIELD \| CONSTR \| METHOD |  |


---


## sbrt.ext.mma.algebra Class MMaExprInterpretter

```
java.lang.Object
  sbrt.ext.mma.algebra.MMaExprInterpretter
```

---

``` public class MMaExprInterpretter extends java.lang.Object ```

This class is intended for use solely by the class `MathematicaSolver`.

**Author:**
:   This class was written and documented by Jeremiah Wright while in the
    Wagner lab.

---

| **Method Summary** | |
| --- | --- |
| `protected static java.util.Map<java.lang.String,LinearComb<java.lang.String>>` | `interpretSolution(com.wolfram.jlink.Expr solution)`             Converts the provided Mathematica expression to a `Map`. |

**Methods inherited from class java.lang.Object**

`clone, equals, finalize, getClass, hashCode, notify, notifyAll, toString, wait, wait, wait`

**Method Detail**

### interpretSolution

```
protected static java.util.Map<java.lang.String,LinearComb<java.lang.String>> interpretSolution(com.wolfram.jlink.Expr solution)
```

:   Converts the provided Mathematica expression to a `Map`.

    :   **Parameters:**: `solution` - the solution to a system of linear equations. **Returns:**: a `Map` representation of the provided `Expr`.


---


|  |  |  |
| --- | --- | --- |
| **Overview** \| **Package** \| **Class** \| **Use** \| **Tree** \| **Deprecated** \| **Index** \| **Help** | |  |
| **PREV CLASS**   NEXT CLASS | **FRAMES**    **NO FRAMES**     **All Classes** |  |
| SUMMARY: NESTED \| FIELD \| CONSTR \| METHOD | DETAIL: FIELD \| CONSTR \| METHOD |  |


---
